# Current Status of Tissue Engineering in the Management of Severe Hypospadias

**DOI:** 10.3389/fped.2017.00283

**Published:** 2018-01-22

**Authors:** Tariq O. Abbas, Elsadig Mahdi, Anwarul Hasan, Abdulla AlAnsari, Cristian Pablo Pennisi

**Affiliations:** ^1^Department of Health Science and Technology, Faculty of Medicine, Aalborg University, Aalborg, Denmark; ^2^Department of Pediatric Surgery and Urology, Hamad General Hospital, Doha, Qatar; ^3^College of Medicine, Qatar University, Doha, Qatar; ^4^Department of Mechanical and Industrial Engineering, Qatar University, Doha, Qatar; ^5^Department of Urology, Hamad Medical Corporation, Doha, Qatar

**Keywords:** hypospadias, urethra, urethroplasty, postoperative complications, tissue engineering, biomaterials

## Abstract

Hypospadias, characterized by misplacement of the urinary meatus in the lower side of the penis, is a frequent birth defect in male children. Because of the huge variation in the anatomic presentation of hypospadias, no single urethroplasty procedure is suitable for all situations. Hence, many surgical techniques have emerged to address the shortage of tissues required to bridge the gap in the urethra particularly in the severe forms of hypospadias. However, the rate of postoperative complications of currently available surgical procedures reaches up to one-fourth of the patients having severe hypospadias. Moreover, these urethroplasty techniques are technically demanding and require considerable surgical experience. These limitations have fueled the development of novel tissue engineering techniques that aim to simplify the surgical procedures and to reduce the rate of complications. Several types of biomaterials have been considered for urethral repair, including synthetic and natural polymers, which in some cases have been seeded with cells prior to implantation. These methods have been tested in preclinical and clinical studies, with variable degrees of success. This review describes the different urethral tissue engineering methodologies, with focus on the approaches used for the treatment of hypospadias. At present, despite many significant advances, the search for a suitable tissue engineering approach for use in routine clinical applications continues.

## Introduction

Hypospadias is a frequent genitourinary congenital malformation with an incidence of around 1 per 300 male newborns, although its frequency varies among populations from 0.3 to 7.0 per 1,000 live births (1, 2). Hypospadias results from the malposition of the urinary meatus on the ventral aspect of the penis following incomplete closure of the urethral folds during early gestational weeks. This condition has been associated with hereditary and/or environmental factors that have not been completely identified yet (3). Three abnormalities that are most frequently associated with hypospadias are an abnormal location of the meatus, a curvature of the penis (chordee), and an incomplete ventral prepuce. In general, severe forms of hypospadias are associated with a significant chordee and a urethral meatus located proximal to the mid-shaft of the penis. The ventral axis components of the penis may also display abnormalities, including atrophy of the cavernous corpora and stiffening of the corpus spongiosum (4). Advances in microsurgical instrumentation, imaging devices, suture materials, and reconstructive techniques have significantly improved the outcome of hypospadias surgical repair during the last decade. However, the current management techniques for proximal hypospadias still present several limitations, which are mainly associated with the highly demanding surgical procedures and the graft donor site morbidity. Moreover, complication and reoperation rates remain unacceptably high. These constraints have initiated a drive toward the development of tissue engineering approaches that may offer a better alternative for pediatric urethral reconstruction. In this review, we summarize the limitations of the current surgical management of severe hypospadias and examine the recent tissue engineering developments toward repair of the urethra, with special focus on the research targeted to severe hypospadias in children.

## Current Management of Severe Hypospadias and Its Limitations

Hundreds of different urethroplasty techniques have been reported for hyspospadias repair over the years (5). Treatment of distal hypospadias with one-stage urethroplasty approaches, such as onlay preputial island flaps or tubularized incised plate (TIP) repair, is currently associated with a relatively high success rate. However, management of severe hypospadias remains a challenge, as no single technique is applicable to all patients. There is still an ongoing discussion whether it is most appropriate to perform a single-stage or a two-stage approach. Recent reports indicate that more patients appear to develop complications following single-stage procedures (6) and that staged procedures may result in overall better functional outcomes (7). Yet, retrospective studies of patients undergoing two-stage repair have evidenced complication rates ranging from 50 to 68% (8, 9). The frequency of postoperative complications appears to increase with the anatomic severity of hypospadias no matter what surgical technique is employed (6). The most frequent complications include fistulas and urethral strictures, but can also include dehiscence of the repair or recurrence of chordee.

The outcome of the urethroplasty not only depends on the quality of the anatomical structures and the surgical approach but also on the availability of an appropriate source for the graft, since patients with severe hypospadias frequently require extra tissue to restore the missing urethra. Autologous sources of grafts utilized for urethral replacement include skin from genital areas or extra-genital regions (10–12). These grafts have been superseded by buccal mucosa free skin grafts which is currently the most widely used source (13). Harvesting of oral mucosal grafts represents an easy procedure, causing minimal discomfort for the patient and an acceptable degree of morbidity (14). However, the amounts of tissue available for harvesting are limited and complications may appear, including donor site bleeding, infection, pain, parotid duct injury, graft contracture, and numbness (15).

Further complexities may arise following the pubertal period, in that a failed neo-urethra may result in a scarcity of hair-free surrounding skin for urethral replacement. Moreover, a surgically constructed neo-urethra may fail to develop along with the penis as the affected person matures to post-pubertal age, causing stricture and secondary chordee. Balanitis xerotica obliterans, resulting from the use of adjacent affected urethral or penile tissues for repair, may also cause recurrent urethral strictures in patients who had undergone previous surgery for hypospadias. Eventually, any form of substitution urethroplasty seems to worsen over time, as complication rates appear to increase with the duration of the follow-up (16).

## The Rise of Urethral Tissue Engineering

**Figure 1:**
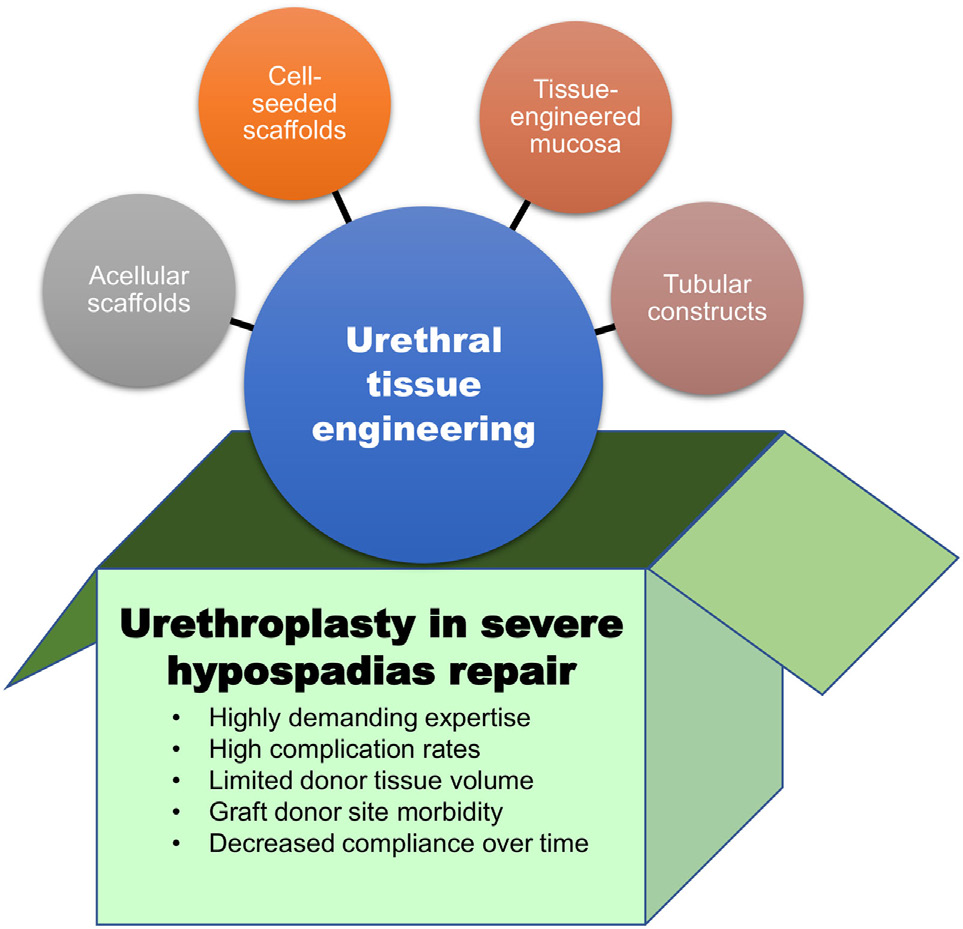
The box contains the main limitations of current surgical approaches for hypospadias repair. Outside the box, urethral tissue engineering approaches that may represent valuable therapeutic options for children with severe hypospadias.

Overall, the disappointingly low success rates, the complications related to graft harvesting, and the tendency of grafts to deteriorate over time, are prompting us “to think outside the box” (Figure 1). Tissue engineering may hold the key to finding novel techniques and tissue sources to replace the missing urethra. Tissue-engineered grafts could be tailored with characteristics like those of urethral mucosa, but conveniently available “off the shelf.” Ideally, tissue-engineered constructs for urethral replacement should be biocompatible, able to be well vascularized and biodegradable (17, 18). The biodegradation process should follow with the regeneration timeframe of the local surrounding tissues to allow the generation of a fully differentiated functional urothelium (19). Moreover, optimal constructs should also be compliant enough to accommodate jets of propelled urine during voiding. Neo-urethral compliance can be adjusted by manipulating the mechanical properties of constructs to optimize stretch functionality (20). Furthermore, the construct should be impermeable to urine, as urine is cytotoxic to surrounding tissues (21).

To overcome the difficulties associated with current urethral repair techniques, during the last couple of decades, extensive research has been performed to investigate biomaterials and cells that could be used alone or in combination for urethral replacement. Table 1 provides some examples of the diverse types of biomaterials and cells that have been assessed for urethral tissue engineering. These sources will be described and discussed in the following sections.

**Table 1:** Examples of biomaterials and cells that have been investigated for urethral tissue engineering.

Examples	Reference
**Synthetic biomaterial scaffolds**	
Polyethylene terephthalate	(23)
Poly (l-lactic acid)-co-poly-(ε-caprolactone) (PLLCL)	(26)
Polylactic acid/PLLCL composite	(27)
**Natural biomaterial scaffolds**	
Collagen type I and III	(28)
Silk fibroin	(31, 61, 62)
Small intestine submucosa (SIS)	(35–37)
Decellularized human amniotic membrane	(39)
Decellularized urinary bladder	(34)
Decellularized porcine dermis	(38)
**Cells**	
Urine-derived stem cells	(48)
Urothelial cells derived from bladder washes	(45, 46)
Adipose-derived stromal cells	(57, 58)
Oral keratinocytes	(63)

## Polymeric and Extracellular Matrix (ECM)-Derived Scaffolds

The scaffold acts as a supporting skeleton for tissue regeneration, maintaining the structural balance of the regenerating tissue and allowing its three-dimensional (3D) regeneration. The scaffolds can be categorized according to their biodegradability (non-biodegradable or biodegradable) or according to their source (synthetic, naturally derived, or a combination of both) (22).

Some of the synthetic non-degradable polymers that have been explored for urinary tract reconstruction include polytetrafluoroethylene and poly(ethylene terephthalate). These materials do not readily promote cellular attachment and, therefore, require a surface treatment to allow for urothelial cell adhesion (23). In general, non-degradable materials used in urinary tract reconstruction have been mainly used as temporary supports in specific clinical scenarios, as they have in general exhibited poor results, with the occurrence of complications including calcification, fistulae, chronic hematuria, encrustation, migration, and significant shortening (24, 25).

Synthetic biodegradable scaffolds, on the other hand, include those made of polymers, including poly-l-lactide (PLL), polycaprolactone (PCL), and poly(lactic-co-glycolic acid) (PLGA). The mechanical properties of these polymers, such as porosity and degradation rate, can be easily tailored to satisfy the requirements of a urethral construct. Polymeric composite membranes fabricated using PLL–PCL have demonstrated to possess mechanical properties suitable for urothelial tissue engineering, supporting growth and phenotype maintenance of human urothelial cells (26, 27). Synthetic biomaterials, however, usually require surface treatments to promote cell attachment, as they lack specific molecular elements for interaction with cells and proteins (22).

Natural polymers, on the other hand, exhibit specific cell adhesion ligands that favor the attachment and growth of cells onto their surface. Different naturally derived biomaterials have been explored for urethral replacement, including collagen (28), hyaluronic acid derivatives, alginate, and chitosan (29). Another promising biomaterial is silk fibroin (SF), a natural polymer obtained from *Bombyx mori* cocoons. This material has shown excellent biocompatibility, with reduced immunogenicity and fewer inflammatory reactions as compared to other biological materials. The mechanical characteristics of SF, including its elasticity and shape memory, were found to be well suited for urologic tissue engineering applications (30). Different cell types relevant for urethral reconstruction have been successfully grown onto porous SF scaffolds, including keratinocytes and fibroblasts (31). SF can also be blend with a synthetic polymer, such as PCL, to facilitate the fabrication of electrospun nanofiber allowing successful growth of oral mucosal epithelial (EP) cells (32).

Extracellular matrix obtained after chemical decellularization of xenogeneic or allogeneic tissues has also been extensively investigated as a scaffold for urethral reconstruction. ECM scaffolds exhibit very rapid biodegradability *in vivo*, releasing degradation components that orchestrate healing and regeneration through a process known as constructive tissue remodeling (33). Decellularized tissues retain most of the structural collagens and proteoglycans, which contribute not only to preserve the structural integrity but also to retain bioactive growth factors promoting ingrowth of endothelial and smooth muscle cells (SMCs) (34). Matrices prepared from small intestine submucosa (SIS) are those that have been most frequently investigated for urethral repair, thanks to their high content of collagens, fibronectin, elastin, and glycosaminoglycans (35–37). Other acellular matrices include decellularized porcine dermis (38), human amniotic membrane (39), and urinary bladder (40). A promising new source for generation of autologous tissue-engineered constructs is decellularized ECM obtained from cultures of progenitor cells (41, 42). These *in vitro* cell-derived matrices might be prepared using urethral-specific cells to provide a mixture of specific ECM components and biological factors that may provide the environmental signals controlling the fate of the diverse types of cells comprising the urethral tissue.

## Cell-Seeded Scaffolds

No consensus has yet been reached on the potential beneficial effects of cell seeding of tissue-engineered scaffolds for use in the urogenital system, although it appears that cells are required for urethral repair of defects that are >0.5 cm in length (43). Although cells harvested from the urinary tract may be the optimal choice for urethral tissue engineering, other sources may be effective as well. A recent study was performed to determine whether EP cells from urinary and non-urinary sources may behave different in terms of their clonogenic capacity and ability to proliferate. Since only few differences were observed between oral mucosal and urethral cells, the authors suggested that these two sources may be similarly efficient to generate stratified epithelium for urethral reconstruction (44).

Several studies have examined the effects of seeding scaffolds with urothelial cells, which may be obtained invasively or non-invasively. Disadvantages of invasive techniques (e.g., open bladder biopsy) include the harvesting of an inadequate number of cells and their requirement for general anesthesia. Moreover, the associated morbidity of the donor site can lead to significant risks, including bleeding and infection. Alternatively, various non-invasive methods have been used to harvest the needed cells as well. For example, urothelial cells have been obtained from bladder washings, a method that has shown to be safe and highly reproducible in adults and children (45, 46). Urine-derived stem cells have also shown the ability to expand and to differentiate into both urothelial and smooth muscle phenotypes (47, 48). Cell proliferation is markedly influenced by the mechanical properties of the polymer scaffolds, and coculture of several cell types was shown to be superior to culture of individual cell types (38), perhaps because the former represents a more physiological condition, involving paracrine signaling among different cell types.

## Fabrication and Addition of Bioactive Factors

**Figure 2:**
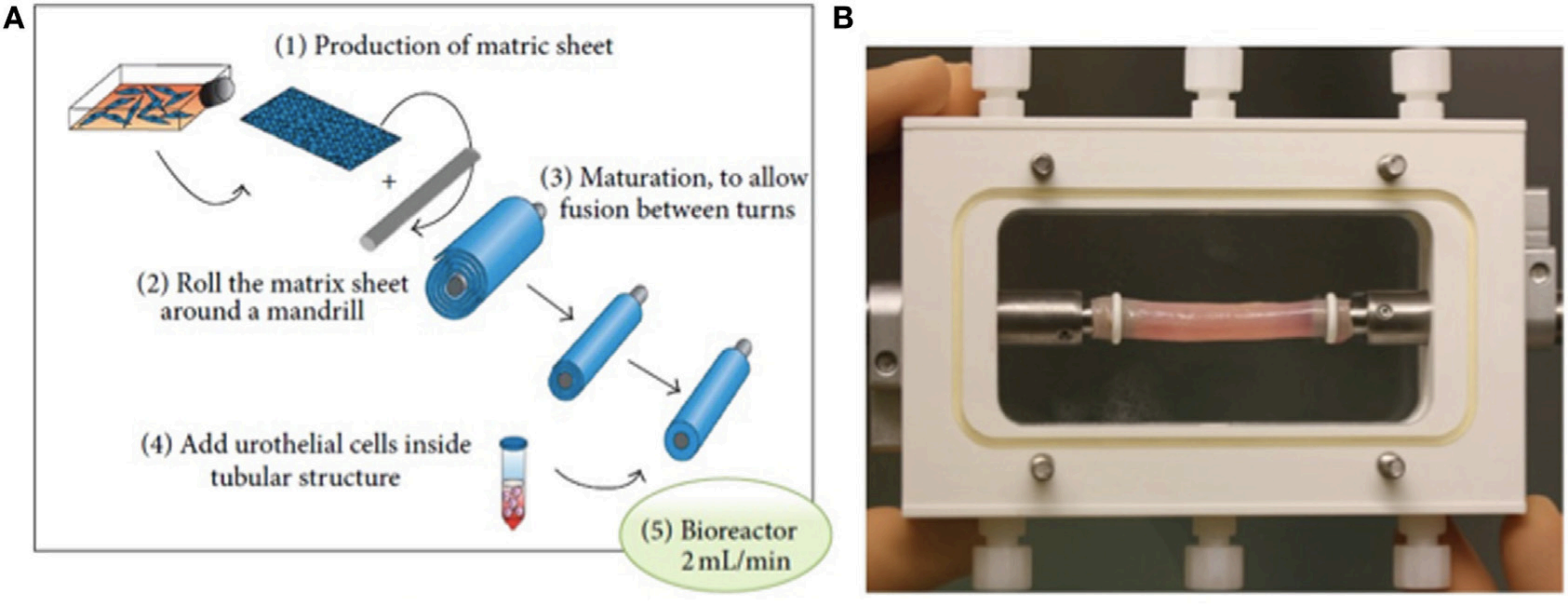
Fabrication of urethral scaffold tubes: **(A)** A matrix sheet of fibroblasts is rolled to form a tube, and urothelial cells are seeded in the lumen. **(B)** Illustration of a bioreactor for tubular cell-seeded grafts to stimulate differentiation and formation of a watertight mucosal layer [reprinted with permission from Ref. (49)]. © 2013 by Orabi et al.

Although several approaches have been utilized to fabricate these scaffolds, an attractive method of fabricating scaffolds for urethral replacement consists of producing cell-seeded tubular grafts that self-assemble (Figure 2) (49). Optimal results require adjusting the mechanical properties of these constructs, including surface topography that is important for ideal growth and differentiation of the cells. For example, attachment of cellular proteins important for urothelial cell adhesion can be enhanced by increasing the external roughness of these scaffolds (50, 51). Scaffold porosity is another parameter that needs to be controlled during fabrication to offer a route for the diffusion of nutrients to the cells, as well as for the elimination of metabolic products (52, 53). Exogenous trophic factors, including natural ECM proteins and growth factors, have been used to bio-functionalize scaffolds creating a microenvironment that simulates the integration of the tissue-engineered constructs (54–56).

## Recent Translational and Clinical Studies Toward Tissue-Engineered Urethral Replacement

Several engineered urethral substitutes have been examined in animal models over the past three decades. Although some studies have demonstrated positive results, the experiments were performed in small series with short follow-up. Notwithstanding, the results of these animal studies have yielded several important conclusions. For example, failure of a urothelial layer to develop on the internal surface of the implanted constructs resulted in leakage of urine, with associated inflammation and fibrosis, leading to re-stricturing. Moreover, seeded constructs seem necessary to treat longer strictures, because the survival of these constructs does not depend on the ingrowth of EP cells from the surrounding healthy tissues but mainly on the pre-seeded cells (49). Efforts to identify better cell sources to seed the constructs have led to the use of adipose stem cells to reconstruct urinary tract epithelium (24, 57, 58) and SMCs (59) in urethral tissue engineering. A recent systematic review showed that, regardless of the type of scaffold material, cell seeding significantly reduced the risk of morbidities. Moreover, the addition of cells reduced failure rates when inlay or full urethroplasties were performed (60).

**Figure 3:**
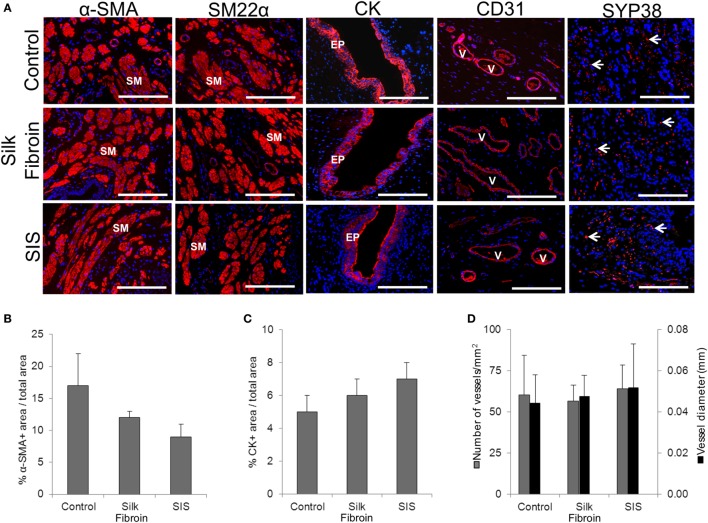
Urethral tissue regeneration following implantation of acellular silk fibroin scaffolds in rabbits. **(A)** Immunohistochemical assays showing the expression of smooth muscle (SM) contractile markers; epithelial (EP)-associated cytokeratins (CK); and endothelial markers. V indicates blood vessels and arrows denote cells of neuronal lineages. Scale bars denote 200 µm in all panels. Panels **(B–D)** display histomorphometric data from alpha-smooth muscle actin positive (a-SMA+) regions **(B)**, CK positive cells **(C)**, and CD31 positive vessels **(D)** obtained from control and scaffold implanted animals. [reprinted with permission from Ref. (61)]. © 2014 by Chung et al.

On the other hand, extent of vascularization represents a key factor for the success of the implanted constructs. In this direction, vascularization has been analyzed immunohistochemically in a rabbit model following ventral onlay grafts of SF (Figure 3). The samples of tissue-engineered urethra revealed SMC differentiation, EP maturation, and *de novo* vascularization and innervation processes (61). Several other preclinical studies strongly support the use of SF as a material for urethral tissue engineering, showing improved vascularization and reduced immunogenicity in comparison to conventional SIS scaffolds (61, 62).

When considering the type of the reconstruction and the related shape of the construct, animal studies showed that synthetic materials did not perform as well for inlay repair as compared to full repair (60). It is also desirable to produce an artificial graft with characteristics similar to those of buccal mucosa and a tissue-engineered buccal mucosa for urethral replacement showed optimal short-term results in a rabbit model (63). Long-term results, on the other hand, showed that the collagen fibers in the construct were significantly more disordered than those in normal urethral submucosa (64).

Intriguingly, a recent meta-analysis comparing outcomes of several urethral tissue engineering preclinical and clinical studies has revealed that the favorable results seen in animal models are not always translated into the patients (60). A feasible explanation for these differences could be the fact that preclinical studies use animal models with a healthy urethra, limiting the level of evidence provided by the animal model.

Concerning clinical trials, several studies have been carried out to assess the performance of tissue-engineered grafts in pediatric patients (summarized in Table 2). In general, results using scaffolds or tissue-engineered constructs have been suboptimal in patients who had preceding failed urethroplasties and those with unhealthy vascular beds (65).

**Table 2:** Outcomes of clinical studies of tissue-engineered urethral replacement in pediatric patients [adapted from Ref. (15, 60)].

Material	Approach	Number of patients	Age	Follow-up	Outcome	Reference
Collagen-based matrix	O	4	4–20 years	22 months	Three patients had successful cosmetic and functional outcomes	(13)
Gelatin sponge	–	8	8–36 months	12 months	Implants were successful in all patients	(66)
Acellular skin, human	C	8	4–23 years	4–6 months	Implants were similarly successful in all patients	(40)
PGA and PLGA	C	5	10–14 years	36–76 months	Implants were successful in all patients	(67)
Acellular skin, human	O	6	14–44 months	6–8 years	All patients had good functional and cosmetic results	(70)
SIS, 4-layer, porcine	O	12	1.5–15 years	6–36 months	Implants were successful. Three patients developed fistulas and failure of the graft	(71)

*O, onlay; C, circumferential; PGA, polyglycolic acid; PLGA, poly(lactide-co-glycolic acid); SIS, small intestine submucosa*.

In 1999, Atala et al. showed that the use of bladder submucosa and a collagen-based inert matrix for urethroplasty in four patients with hypospadias yielded positive results (13). Despite the small number of patients, the short duration of follow-up (22 months) and the development of a fistula in one patient, these findings suggest that tissue-engineered materials should be tested in larger numbers of patients. 10 years later, use of a porous gelatin scaffold and preputial mucosa or a urethral plate graft combined with local flap to repair severe hypospadias in eight patients revealed that all were successful after 1-year follow-up (66).

A porous gelatin scaffold was also shown to be successful in eight patients with hypospadias, of mean age 13 years in a consecutive experiment (40). Another clinical trial, in which patients were followed up for 6 years, found that tubularized constructs composed of PGA:PLGA meshes seeded with bladder cells were successful in managing urethral trauma in pediatric patients (67). However, degradation products of polyester-based scaffolds can induce chronic inflammation *in vivo* (68) and may, therefore, adversely affect the long-term fate of implanted constructs (69).

In 2013, Fossum et al. tested the long-term (about 8 years) effects of cultured autologous urothelial cell implants in hypospadias patients (70). That study found that use of these implants in patients with severe hypospadias resulted in high complication rates, but with results equal to or better than expected for their phenotypes. However, the limitations of this study included the small number of patients and the absence of a control group.

**Figure 4:**
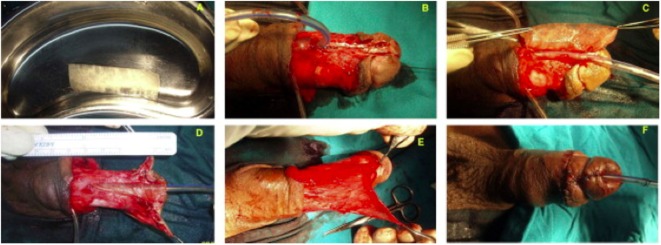
**(A)** Gross appearance of a small intestine submucosa (SIS) graft; **(B)** penile skin degloving through a subcoronal incision, preserving the urethral plate; **(C)** suturing of the SIS graft in an onlay fashion; **(D)** suturing of the completed onlay SIS graft; **(E)** splitting of the dartos flap into two halves to form the second layer of coverage for the graft; and **(F)** final postoperative appearance [reprinted with permission from Ref. (71)]. © 2013 by Elsevier B.V.

A recent clinical study found that SIS grafts were successful in circumcised patients with hypospadias or undergoing repeat hypospadias repair (71) (Figure 4). Three of the 12 patients developed fistula, which may have been caused by the occurrence of infection, but these fistulas were easy to repair. Limitations included the heterogeneity of the hypospadias (distal, mid-shaft, and proximal), the heterogeneity of patient age (from infants to teenagers), and the inclusion of untreated patients and those who had failed previous repair, all of which might generate confusion in the interpretation of results.

## Conclusion and Future Directions

Current urethroplasty techniques for the management of severe hypospadias are associated with significant morbidities and limitations that strongly mandates the need to “think outside the box.” Advances in urethral tissue engineering biomaterials and fabrication methods have built a solid base on which future urethral replacement approaches can stand on. The currently available experimental evidence suggests that long and complex urethral defects would require tubular cell loaded constructs in contrast to onlay grafts, which have shown benefit in shorter defects. Although the search for an “ideal” combination of biomaterials and cells for urethral replacement continues, it appears that optimal results could be obtained with composite materials seeded what different cell types (stromal and EP). Future research in this field should take into consideration more realistic animal models to bridge the translational gap between animal models and clinical studies and the importance of longer follow-up periods. We believe that biodegradability is a crucial factor for successful urethral tissue-engineered regeneration in children, which may accompany the natural growth of penile size at puberty.

## Author Contributions

TA conceived the article, planned and prepared its structure, and performed the bibliographical search. TA wrote the manuscript draft under supervision of CP. EM, AA, AH, and CP edited sections of the manuscript and contributed to the critical revision of the final draft.

## Conflict of Interest Statement

The authors declare that the research was conducted in the absence of any commercial or financial relationships that could be construed as a potential conflict of interest.
